# Older people living at home: experiences of healthy ageing

**DOI:** 10.1017/S1463423621000049

**Published:** 2021-03-04

**Authors:** Astrid Fjell, Kristin Ådnøy Eriksen, Monica Hermann, Anne-Marie Boström, Seiger Berit Cronfalk

**Affiliations:** 1 Department of Neurobiology, Care Sciences and Society, Division of Nursing, Karolinska Institutet, Stockholm, Sweden; 2 Department of Health and Caring Sciences, Western Norway University of Applied Sciences, Bjørnsonsgate 45, 5528 Haugesund, Norway; 3 Department of Health and Caring Sciences, Western Norway University of Applied Sciences, Klingenbergvegen 4, 5414 Stord, Norway; 4 Department of Neurobiology, Care Sciences and Society, Division of Nursing, Karolinska Institutet, Stockholm, and Theme Ageing, Karolinska University Hospital, Alfreds Nobels Allé 23, 141 83 Huddinge, Sweden; 5 Department of Health Sciences, Red Cross University College, Department of Neurobiology, Care Sciences and Society, Division of Nursing, Karolinska Institutet, Alfreds Nobels Allé 21, 14157 Huddinge, Stockholm, Sweden

**Keywords:** age-friendly environment, content analysis, focus group, healthy ageing, public health, social support

## Abstract

**Aim:**

The purpose of this study was to investigate how old persons perceived their life to be, how they viewed the ageing process and their need of health care and societal support.

**Background:**

The purpose of WHO’s Healthy Ageing strategy and development of age-friendly environments is to support physiological and psychosocial changes in old persons by facilitating basic needs. Interventions to operationalize these needs in older people living at home are often developed from a professional perspective and to a small extent involves the perceptions, experience and expectations of the older persons.

**Method:**

This qualitative study has an explorative design using focus group discussions to collect data. In all, 34 persons between 69 and 93 years of age participated in seven group discussions. The interviews were analyzed using inductive manifest content analysis.

**Findings:**

The main results suggest that most old persons enjoyed life and wished it to continue for as long as possible. Important was to sustain networks and to feel useful. Unexpected changes were described as threats and the need to use health care services was associated with illness and being dependent. The result is presented in three categories with sub-categories: ‘Embracing life’, ‘Dealing with challenges’ and ‘Considering the future’.

## Background

In much of the literature, old age is associated with declining health through illnesses and functional decline (Clegg *et al*., 2013; Morley *et al*., 2013). However, if the added years are characterized by good health, old persons may be able to experience psychosocial changes that will contribute to remained well-being (Steptoe *et al*., 2015; World Health Organization, 2016).

Healthy ageing is promoted by The World Health Organization (WHO) as a strategy on ageing and defined as ‘the process of developing and maintaining the functional ability that enables well-being in older age’ (World Health Organization, 2015). The purpose is to support physiological and psychosocial changes in old persons by facilitating their basic needs (World Health Organization, 2015; Beard *et al*., 2016). One such area of development is an age-friendly environment with the purpose to promote health and support to old people (Beard *et al*., 2016; Klimova *et al*., 2016).

Research suggests that strategies on healthy ageing and the development of age-friendly environments are necessary (Lloyd-Sherlock *et al*., 2012; Dattilo *et al*., 2015). The systems are often developed in specialized contexts (World Health Organization, 2015; Beard *et al*., 2016) and are thereby understood as such, as needs, expectations and opinions are described from the professional’s‘ perspective rather than that of the old persons (Derksen *et al*., 2012; Marcus-Varwijk *et al*., 2016). Lette *et al*. (2017) showed that old persons still living at home had different views about what was important in life compared to that of health care professionals. In addition, health professionals did not think about the old persons perspective and knowledge about ageing (Beard *et al*., 2016; Lette *et al*., 2017; Marcus-Varwijk *et al*., 2019). The lack of knowledge is described by Kontis *et al*. (2017) and Beard *et al*. (2016) who point out the need for knowledge about ageing among professionals in health care and social services.

Hjelle *et al*. (2017) showed that being socially active and taking part in leisure activities were important for old persons living at home. This is in line with Stephens, Breheny and Mansvelt (2015) who suggested a number of perspectives on healthy ageing in six domains: physical comfort, social integration, contribution, security, autonomy and enjoyment.

To further help identify persons at risk of poor health a study regarding preventive home visits (PHV) was performed in the western region of Norway (Cronfalk *et al*., 2017). The purpose was to develop a sustainable model of PHV for home dwellers older than 75 years of age. Persons from two municipalities, in all 257 were approached of which 167 participated. Results from the study showed that every fifth person was identified with increased risk of poor health and developing illness (Cronfalk *et al*., 2017). Based on the importance of welfare provided to old people in Norway, a reform was launched; ‘A full life – all your life’ (White Paper, 2017) especially aimed at the health and care sector, to generate health and care services for all older persons in Norway. The question ‘What is important to you?’ was asked. The reform is adamant that all sectors nationwide must contribute to create an age-friendly society, where old persons can remain active and independent through their whole life. The reform was motivated by the Ministry of Health and Care Services strategy document ‘More Years – More Opportunities – The Norwegian Government’s strategy for an age-friendly society’ (White Paper, 2016). The reform is a strategic 5-year program 2019–2023, with the aim of planning and utilizing Norwegian age-friendly environments. This reform focuses on four key areas of importance: longer working life, age-friendly local communities, the health and care sector and research on ageing. This is in line with WHO‘s strategies on age-friendly environments (World Health Organization, 2015). The purpose of this study was to investigate how old persons perceived their life to be, how they viewed the ageing process and their need of health care and societal support.

## Method and design

This study employed a qualitative approach applying an explorative design using focus groups to collect data. Data were analyzed using inductive content analysis (Graneheim and Lundman, 2004; Graneheim *et al*., 2017).

Focus group discussions are a prearranged semi-structured interview led by a skilled facilitator and should be used when you need to understand an issue at a deeper level than you can access with a survey. They are helpful for adding meaning and understanding to existing knowledge or getting at the ‘why’ and ‘how’ of a topic. Focus group discussions are defined as carefully planned sessions involving several informants within a group. The group is often homogenous to encourage dynamic discussions gaining access to people’s experiences and understanding of specific topics (Polit and Beck, 2017). The discussions are characterized as a process where the interaction between the persons facilitates the sharing and comparing of experiences by spontaneous and emotional statements (Morgan, 1997; McLafferty, 2004; Patton, 2015; Brinkmann and Kvale, 2015). According to Morgan (2010) and Morgan and Bottorff (2010) sharing may contribute to personal growth through the engagement of discussing topics of interest. Focus group discussions are conducted by one facilitator and one observer both with specific responsibilities during the discussion. The facilitator´s role is to lead the discussion by introducing the structure of the conversation, topics and ask broad questions for prompt responses. The observer´s role is to follow the discussions and put forward questions to clarify any misunderstandings or disputes and to present a conclusion. The ideal number of participants differs in the literature. Côte-Arsenault and Morrison-Beedy (1999) suggest that sensitive topics need smaller groups of no more than five persons. Most literature, however, suggest five to eight participants (Patton, 2015).

### Setting and sample

Old persons were recruited from two municipalities, both set in the western part of Norway. The difference between the two was their number of inhabitants as one had approximately 45.000 and the other almost 12.000. Each municipality presented arenas for old residents that is, two activity centers, one in each municipality, all with easy access by local transport facilities.

Inclusion criterion: retired persons residing in their private home and comprehending the Norwegian language (read, write and speak). Exclusion criterion: cognitive impairment affecting the ability to understand the purpose of questions fully.

In all, 34 persons (28 women and six men) accepted participation. All, but six participants resided in the large municipality.

Retired persons attending activity centers and non-government-organization were informed about the study. The activity centers were comprehensive and included both physical and intellectual activities such as group physical activities and training sessions and courses such as cooking and arts and crafts classes, as well as courses how to stop alcohol and smoking addictions. The non-government-organization offered for example language courses. They received written information from the managers at each site. This was followed by an information meeting held at each site by the first and second author. The meeting included information about the purpose of the study and details about the time schedule (date, time and place) as well as the procedure linked to the group discussions. Information was also given about how data (the tape-recorded discussion) would be kept and protected and that each person´s anonymity would be secured. Information that participation was voluntary was also included. Interested persons were requested to contact the first author to provide written consent.

In all, seven focus groups were arranged with three to six participants in each group (see Table 1). The group discussions began with a brief verbal information about the procedure by the first author. The participants were introduced to the principles for focus group discussions such as not interrupting each other and to raise one’s hand to talk. They were informed that the discussion was tape-recorded and that the recording started when the discussions began. At the start of the discussion each participant introduced him/herself by a number, to identify the participants at a later phase and during the analysis. The participants introduced themselves by a number (depending on number of participants in the group). The moderator used a semi-structured interview guide to lead the discussions. Key questions were: *Can you please describe what good ageing is to you? Can you please describe what you do to manage the challenges when they occur?* and *In what way can health care services support you in order to maintain good health?* The questions were followed by the facilitators and probing comments or questions to encourage the participants to further reflect and elaborate their answers and thoughts. In all, three different observers participated at separate times throughout the focus group discussions. The discussions lasted between 65 and 95 min. Notes concerning the participant´s non-verbal expressions were not notified such as facial expressions nor were sights, mumbling or laughter.

**Table 1.** Overview of the participants

Focus group	Total participants	Male	Female	Age	Municipality	Home care services
1	4		4	78–86	Urban	1
2	3	1	2	69–86	Urban	1
3	5		4	75–84	Urban	
4	5	5		75–90	Urban	1
5	6		6	73–93	Urban	1
6	5		5	71–80	Urban	
7	6		6	73–82	Rural	

### Analysis

Data were analyzed using inductive manifest content analysis as described by Graneheim and Lundman (2004) and Graneheim, Lindgren and Lundman (2017). Following the focus group discussions, the first author listened through the recordings to ensure that each question had been addressed and pursued. The tape recordings were then transcribed verbatim by a professional transcriber. This was followed by an initial reading of the transcripts to get an overview of its substance, making notes in the margin about the content of the text. Meaning units were then identified and marked throughout the text. Condensed meaning units were then labeled as codes, closely related to the text. The codes were sorted into groups based on their similarities forming five sub-categories. Categories describing groups of sharing similar content were then identified. In all, three categories were identified, discussed and compared by the authors until consent was reached. The underlying meaning of the categories and sub-categories merged into an overall theme, formed during the process of analysis. (see Table 2).

**Table 2.** Example of the analytic process including the three categories and overall theme

Meaning unit	Sub-category	Category	Theme
‘Indeed, I feel great, because I am healthy and can participate in activities, I can travel, my financial situation is good, my home is centrally situated’.	Health and identity	Embracing life	
‘These things you normally postpone until the day comes, right? When the bomb goes off, then you need to deal with the situation as it is’.	Life challenges	Dealing with challenges	So far so good…
‘My fear is that I will become a burden and not an asset, and that I have to be very conscious that they should live their own lives and not think about how their mother and father are doing first’.	Social support and health care services	Considering the future	

### Ethics

This study was approved by the Norwegian Social Science Data Services A/S NSD (No. 44970).

## Results

The main findings showed that most participants felt confident and enjoyed life to the fullest as they were in good health and were able to retain social relationships. A strong point was keeping their integrity and autonomy. This was predominantly achieved through personal relationships and social networks. Even with a positive outlook on life, some participants expressed worries about the changes and challenges that might occur later in life. Poor health and illness were described as causes for concern as it would inevitably mean, being dependent on others. The results also show that in general, the participants expressed no need for societal support. The results will be presented in three categories: ‘Embracing life’, ‘Dealing with challenges’ and ‘Considering the future’ and three sub-categories and the overall theme, ‘ So far so good … ’, with the implicit meaning that growing old was to enjoy life here and now.

### Embracing life

Most participants described themselves to be in good health and vigor and their main goal was to continue their life as before. This included personal relationships, social networks and sometimes their professional identity. Interactions with people were described as important and influenced their lives positively.

‘Good old age to me means, feeling okey in my head, and doing almost everything I want to do’. (Woman, 81 year).

#### Health and identity

Being healthy, independent and feeling secure financially contributed to the participants´ feelings of confidence while growing old. Below described by three quotations:

‘Indeed, I am great, because I am healthy and can participate in activities, I can travel, my financial situation is good, and my home is located in the central part of the community’ (Woman, 73 years).

‘Good ageing is actually, as long as we are two and we are doing well’. (Woman, 84 years).

‘I can still get up in the morning, care for myself, cook, clean and go for walks, so far old age is ok’. (Woman, 83 years).

Even so, for some their changed identities after retirement was described as a challenging experience, as the profession had been a main part of their identity the whole life. The following quotation exemplifies the importance of professional identity and social relationships that adhere to work.

‘I consider it to be an association between one’s previous experiences in life and one´s professional working life as you grow older and not least, the friendships’. (Male, 76 years).

Other aspects of importance were different personal engagements and networks in the municipalities. These provided the participants with daily structure and routine and devotion to take on important and useful tasks helping others. The ability to socially interact with others also facilitated feelings of security. The social networks were described as stimulating both physically and mentally as the various activities with others reduced feelings of loneliness. The activities offered by the networks were used to maintain interests but also introducing new ones providing the opportunity to meet and make new friends.

‘I still have the opportunity to participate in most activities. And I believe that is important for me to have good healthy ageing. And not least, about friendship, I mean contact, good friends and healthy hobbies. That there is a relation between what you did in earlier years, and what you do now as you are older’. (Male, 76 years).

Activities such as reading, modern technology (computers, tablets and mobile phones, ICT) and other intellectual activities were described as important to keep one´s intellectual abilities intact. Discussions about society and politics were experienced as important, especially in their municipalities.

‘Yes, to still be passionate about politics, and to use the TV to pay attention to what happens in the country, the parliament, the city council, and such. That you could, maybe get a chance to discuss it’. (Male, 86 years).

‘I will point out that being in good health and in addition having a good relationship with the family around me. as well as being part of the society in general and to be able to contribute even as a retired person, is a great feeling’. (Male, 75 years).

The participants also described that their living conditions and housing were important and part of their social life. Several stated that shared housing was relevant for older people, as it would give them varied opportunities and activities within the close vicinity.

‘Something like shared housing, where everybody has their own room and privacy, but also the opportunity to seek out others when they feel like talking to someone, or socialize around meals, maybe a glass of red wine for dinner on Saturday. This has to do with quality of life no matter what kind of situation you are in’. (Woman, 69 years).

### Dealing with challenges

Aware of life´s uncertainties it was still important to live life as best one could and adapt to changes and challenges when they occurred. The participants described age and ageing as dealing with a challenge when life itself was in transformation.

#### Threats and challenges

To be in good or decent health was emphasized as important in order to experience good ageing. Health was however, considered as something that quickly could change their lives at any time. Some expressed fears about dementia, use of medication and loss of driver’s license due to old age or illness. These aspects were described as a threat that could challenge and change their lives as it may prevent them from living the life they wished for.

Some participants described that they took life as it comes, still somehow assuming that ‘something’ would happen.

‘When the bomb goes off, then you need to deal with the situation as it is’. (Male, 83 years).

Some participants described experiences of how age affected them and that they needed to make changes suitable for them. An example was to change from a skiing holiday in the mountains to visiting a hotel, watching the skiers. The participants described this as their own responsibility in order to live the life they sought. Still, most participants did not waste any time thinking about their future. They said that they would face the problems as and when they occurred.

‘I don’t feel old at all, and I guess that has to do with health, and the circumstances of life now. But disaster can happen anytime, especially at our age’. (Male, 83 years).

‘When people die and disappear, no one will fill the space they had. I think that it is sad and when you lose someone you feel exposed or unprotected in a sense. And it is not easy to find new friends, but I am lucky to have a solid network’. (Woman, 84 years).

The fear of how to deal with challenges concerning current and modern technology was discussed and for some it was experienced as an inconvenience and sometimes a threat.

«…We talked about it (for example phoning health care services), that it is scary and we get nervous. Then you pick up the phone, and the voice says; ‘you now get 4 options, you now get 5 options, and everything happens so quickly that it is impossible to write down the number (several mumbles in agreement). It really scares me, that you need to be so self-reliant, and when you try, it is not easy. No, I think a lot of people will agree with me that it is not easy, and that’s what I find scary’. (Woman, 84 years).

### Considering the future

Even though age-related illness was distant for most participants, some expressed concerns about losing control and not being able to manage their own lives.

#### Social support and health care services

For some fear of not being able to cope with every-day-life caused feelings of distress as they did not want to become a burden or dependent of others. The participants described that they could see themselves getting help from the family members, even if it would be difficult.

‘My fear is that I will become a burden and not an asset, and I am very conscious that they should live their own lives and not think about how their mother and father are doing’. (Woman, 70 years).

A common reflection among the participants regarded the services offered by the municipality as they considered it to be of poor quality and home care services were mediocre due to lack of staff and time to carry out appropriate care. The participants described how being dependent on health care services also included the assumption that help was so much more than just practical hands-on help with personal hygiene. It included personal aspects such as care to manage lack of intellectual abilities and to help maintain social networks. Some described concerns about being left on their own when they needed help. Another aspect was that the health care services in general showed poor respect for old people. They described the current health care services as being formed by the notion that people in general should have knowledge about their own health or illnesses and also be  capable to claim thier own rights.

‘I feel that they (health care services) take it more lightly when you are old. So, you

must be pushy, if you feel that you must. You can’t take it for granted that you will

get the follow-up that you got when you were younger’. (Woman, 69 years).

Some participants wished to live at home for as long as possible provided that when they needed the health care service it was good. Others were comfortable with the thought of moving to a residential home as they did not want help from the home care services.

The participants´ main concern was to feel secure. For this to happen they needed to know who to contact. The most common request concerned services from physiotherapy and occupational therapy to assist with tools and equipment in their homes related to ill health or disabilities.

‘If my health should require help with that, I would like the public health care system to give me such services. That I can get out and maintain contact with others’. (Male, 86).

### ‘So far so good …’

The overall theme derived from the three categories suggested that old persons in our study wanted to pursue an independent life, maintain their identity, relationships and social networks for as long as possible. Although they did realize that life would change, it was not something they spent time thinking about. Their wish and ability to lead a full and balanced life close to family, friends, nature and activities were important for their feelings of well-being. To help them, modern technology (ICT) was used and regarded as positive. Still, some pointed out the risk of it replacing human relationships and thereby increase feelings of loneliness among the older generation.

## Discussion

The results of this study were merged data collected from participants in the Western part of Norway with the aim to investigate how persons perceived life as growing old. The results show that old people in this region, shared experiences of what a healthy life and ageing involved. The results agree with previous studies (Stephens *et al*., 2015; Steptoe *et al*., 2015; Hjelle *et al*., 2017) suggesting that growing old and healthy ageing are associated with how a person live his or her life, rather than where. The perspective of being in a social context such as attending an activity center was described as important. It meant that the participants could satisfy their social, physical and mental needs.

In the present study, the participants described personal relationships, maintaining their identity and social networks as important. The importance of being able to continue life as they knew it was appreciated. The social context and interactions with others influenced their lives positively and was described as essential. It meant being part of different social activities and being able to see family members and friends. Their social context being central to this view is shared with Garbaccio *et al*. (2018). Studies by Garbaccio *et al*. (2018) and Halaweh *et al*. (2018) identified important factors that are in line with our results. For example, financial independency, having a purpose in life, being able to stay in the private home, enjoying social activities in the community as well as enjoying relationships with family and friends.

Well-being is described by Kunzmann, Little and Smith (2000) as generally involving feeling happy, being engaged in relationships, continued sense of autonomy and implies the persons own feelings related to security and comfort. According to McNulty and Fincham (2012) the phenomena of well-being can be understood as a relationship or joint experience that can hold both positive and negative feelings and experiences. The sense of well-being is then determined by the relationship between the persons´ different qualities, attributes and shared context. In the present study the feeling of being healthy or in good health that is, no significant health-related problems was described as crucial for the person’s well-being and agrees with Van Leeuwen *et al*. (2019) who suggests that being healthy is important to keep good quality of life when growing old. Halaweh *et al*. (2018) did also find that staying healthy and free from illness was considered important in order to age well.

The present study also presents aspects of using various modern technologies (ICT). The participants were divided in their opinion and interest in using ICT. Some were concerned that it would increase the feeling of loneliness rather than being of help. Others meant that it was valuable as the possibility to keep in contact with family and friends as well as increased gaining of information in general. Some expressed their worries of not being able to understand or comprehend the new technology. According to Baker *et al*. (2017), who investigated a population of old people in rural districts, individuals with no previous experience of ICT use were able to adapt to an ICT tool. These results are in line with Klimova *et al*. (2016) who identified that being active using ICT devices among old people could prolong life. Although Klimova *et al*. (2016) results are mainly positive, it is suggested that not all old persons want to have the means or support to interact with ICT use. This is in coherence with the results from the present study as feelings of not being comfortable in this regard were uttered by some. In order to meet the increased demands for ICT use, societies need to provide technological services such as training programs in order to help facilitate the use of ICT.

Even though the imminent threat of illness was described as distant for most participants, some did express concerns. The fear of losing control in life due to illness such as dementia and thereby being a burden to family members and friends was a daunting perspective. This would eventually mean that they most certainly would need support from social services which was frightening. The preconception was that the services delivered poor quality care and a common reflection was that the home care services were mediocre. Halaweh *et al*. (2018) suggested that understanding the social structure and knowing how to access health care services are important. The participants in our study were well aware of the available health care services but described doubts about using them. This was mostly due to bad experiences or information from others. This is in line with findings of Cronfalk *et al*. (2017) describing preventive home visits (PHV) as a tool to identify old person’s health and living conditions in Norway. Those results indicated that nearly half of the population eligible to participate did not accept the invitation to take part in the study. An explanation for this may be obviated in the present study as the participants portrayed their perspective of PHV as both unnecessary and partly offensive, mainly since the persons offered to participate in PHV all considered themselves healthy and in no need of support. This could arguably be a predicament, as Norway and other western countries tend to recommend PHV to detect early risks of illness and functional disability among the growing population of older people (Vass *et al*., 2007; Behm *et al*., 2013; Luck *et al*., 2013; Cronfalk *et al*., 2017).

According to Halaweh *et al*. (2018) health care services need to consider how to best promote their support and strategies to meet the need of older adults. This is shared by Garbaccio *et al*. (2018) who also argue that it is pivotal for professionals working in health care services to have knowledge about how older people perceive their quality of life. This is in line with our results as the participants suggest that staff working at home care services and health services generally have poor knowledge and therefore may have difficulties to meet the person’s individual needs. Another aspect of importance was how each person understood the future and what would come. Some told about their ability to set aside worries as they took life in their own stride adapting to challenges as they occurred. The need to feel and be independent was crucial and has also been described by Van Leeuwen *et al*. (2019) and Halaweh *et al*. (2018) showing that being independent allowed older people to feel free, enjoy a fulfilled life and hence, improve their quality of life. Healthy ageing is also described as associated to each person´s responsibility regarding their own independency (Marcus-Varwijk *et al*., 2016; Ten Bruggencate *et al*., 2018).

In modern day Norway, as in other countries in the Western world the basic idea is that old persons want to stay at home for as long as possible (Daatland and Veenstra, 2012). This is however, contradicted by Perry (2014) and Munkejord, Eggebø and Schönfelder (2018) as they suggest that old people want to move to shared accommodations with facilities that can contribute to feeling secure and at the same time facilitate their social life. In the present study, some expressed a desire to relocate to shared accommodation as it was perceived as a possibility for a flexible life in a secure environment. Still, most expressed a wish to stay in their private homes for as long as possible. One reason for this may be that the location of social services and activities in their local community was at a close distance. The strategy document from the Norwegian government ‘More Years – More Opportunities – The Norwegian Government’s strategy for an age-friendly society’ (2016) with its key areas and plans to utilize age-friendly environments included the possibility to extend a person´s working life but also a need to further investigate aspects of ageing through research.

The purpose of this study was to explore older person’s experiences of growing old in Norway. The results in our study suggest that old people feel healthy are active and mostly see positively on their future. Their need for support from the health care services was limited and unwanted as they perceived themselves to be in good health.

### Strengths and limitations

This study focused on old persons´ experiences of growing old and ageing. Focus groups were chosen to collect data (Krueger, 2014). The purpose of using groups rather than individual interviews was to facilitate a dynamic discussion among the participants and to share experiences of what growing old incorporated. The shortcomings of choosing focus groups could be the challenge of guiding the discussions. However, in this study a moderator and facilitator were both present during all discussions which was considered a strength. A different aspect of group interview could have been the nominal group technique. Still, its methodology is described by Allen *et al*. (2004) as predominately suited for research that include participation from various sources for example health professionals, patients, relatives or general public. The method involves a different planning schedule including a number of different phases. The process includes repeated surveys to collect data, developing instruments or questionnaires to collect participatory opinions and ideas to share during the group discussion itself. This was not considered in the present study as the aim was to investigate older persons alone, and their experiences of ageing.

Another limitation is the small number of men compared to women that participated. Still, this may reflect the ageing society in Norway where women have a life expectancy of 84 years and men 80 years.

## Conclusions

The results show that being independent and in a social context was important when growing old. The activity centers were important social factors as they facilitated networks and made the participants feel useful and offered structure in life. The opportunity to live close by and to have access to various facilities was considered to affect quality of life and sense of well-being. The results show that being healthy, independent, having meaningful relationships and social activities as well as being financially independent were important to all participants. The results also suggest that health care services were not seen as necessary as long as their health was perceived as good.

## Implications for research and practice

There has been a strong focus on interdisciplinary collaboration within the health care services system, but this collaboration should also include other sectors to build age-friendly environments. Future research should develop models for collaborations between different sectors and evaluate the models´ impact on older persons’ health and well-being. Healthy ageing for many of the participants was to be a part of, but also to live in a social context, which should have consequences for how to plan the community and housing. The participants also expressed concerns about increased use of ICT in both the health care service and in other part of society, which implies that older people should be offered training program to facilitate their use of ICT.
